# Sample-efficient multi-agent reinforcement learning with masked reconstruction

**DOI:** 10.1371/journal.pone.0291545

**Published:** 2023-09-14

**Authors:** Jung In Kim, Young Jae Lee, Jongkook Heo, Jinhyeok Park, Jaehoon Kim, Sae Rin Lim, Jinyong Jeong, Seoung Bum Kim

**Affiliations:** School of Industrial and Management Engineering, Korea University, Seoul, Republic of Korea; Southwest Jiaotong University, CHINA

## Abstract

Deep reinforcement learning (DRL) is a powerful approach that combines reinforcement learning (RL) and deep learning to address complex decision-making problems in high-dimensional environments. Although DRL has been remarkably successful, its low sample efficiency necessitates extensive training times and large amounts of data to learn optimal policies. These limitations are more pronounced in the context of multi-agent reinforcement learning (MARL). To address these limitations, various studies have been conducted to improve DRL. In this study, we propose an approach that combines a masked reconstruction task with QMIX (M-QMIX). By introducing a masked reconstruction task as an auxiliary task, we aim to achieve enhanced sample efficiency—a fundamental limitation of RL in multi-agent systems. Experiments were conducted using the StarCraft II micromanagement benchmark to validate the effectiveness of the proposed method. We used 11 scenarios comprising five easy, three hard, and three very hard scenarios. We particularly focused on using a limited number of time steps for each scenario to demonstrate the improved sample efficiency. Compared to QMIX, the proposed method is superior in eight of the 11 scenarios. These results provide strong evidence that the proposed method is more sample-efficient than QMIX, demonstrating that it effectively addresses the limitations of DRL in multi-agent systems.

## Introduction

Multi-agent reinforcement learning (MARL) is a dynamic and challenging field of study in RL. MARL is concerned with how multiple agents can interact with and influence the environment in a decentralized and cooperative manner, with each agent operating to maximize its own reward while simultaneously considering the actions and goals of other agents within the system. Recently, the study of MARL has gained increasing importance because of the significant growth of complex and interdependent systems. These systems often require cooperation among multiple agents to address various multi-agent problems [1–3] and achieve optimal performance. This could include a wide range of systems, from autonomous robots working together to perform complex tasks, computer networks optimizing data transmission routes, multi-player games engaging interactive play among multiple players [4, 5], and even human social systems such as political coalitions or economic markets [6].

One of the many challenges of MARL is the need to balance the individual objectives of each agent with the collective objectives of the entire system. If each agent were to operate independently, it may lead to a lack of coordination and potentially suboptimal outcomes for the system [7]. To address these challenges, researchers in the field of MARL have developed a range of approaches, such as centralized training with decentralized execution [8–10], decentralized training with decentralized execution, and centralized training with centralized execution. One particular approach that has gained significant traction in recent years is QMIX [11], which uses a centralized value function to learn a joint action-value function that captures the interactions and dependencies between agents. This allows for improved coordination, scalability, and robustness to changes in the number of agents and has been successfully applied to a range of different applications. However, deep reinforcement learning (DRL) methodologies, such as QMIX, require a significant number of interaction samples because of their low sample efficiency [12]. This can be excessively costly in complex real-world environments and block the ability to learn effective policies in complex digital environments [13]. Consequently, a considerable amount of ongoing research is aimed at improving sample efficiency.

In the field of single-agent RL, various methods have been developed to improve sample efficiency in image-based environments. One promising approach is to apply data augmentation techniques to the data used for learning, thereby allowing the agent to adapt and generalize various changes [12, 14]. The second approach uses self-supervised learning as an auxiliary task for reinforcement learning enabling the agent to learn good representations [15–18]. Recently, research has been conducted on utilizing auxiliary tasks that use generative modeling to reconstruct input values [19, 20] or use representations of past information to predict future frames [21, 22]. In addition, there has been active research on using auxiliary tasks to predict the original state representations from observations with spatially and temporally masked pixels to improve the sample efficiency [23]. These approaches facilitate effective representation learning, ensuring the high performance of DRL in image-based environments. However, no research has used auxiliary tasks to improve the sample efficiency in MARL.

In this study, we propose a masked reconstruction task with QMIX (M-QMIX), which is an approach that uses an auxiliary task to improve the sample efficiency in MARL. Among the MARL methods, we used QMIX, which effectively addresses the limitations of traditional MARL [24–27]. The purpose of this study is to improve the sample efficiency by allowing QMIX’s agent network to learn more effective representations using bootstrap your own latent (BYOL) [28]-based mask reconstruction task as an auxiliary task. Therefore, we hypothesize that when agents acquire a meaningful representation of the data derived from their interaction with the environment, they will possess the capability to explore optimal policies efficiently, even in situations with limited data. To validate this hypothesis, we conduct a performance comparison between M-QMIX and QMIX using a limited number of time steps across 11 scenarios in the StarCraft II micromanagement benchmark [29]. Our method demonstrates that not only does the agent learn a better representation, but the sample efficiency is also significantly improved in each scenario. The main contributions of this study are summarized as follows:

1. The proposed method uses a masked reconstruction task as an auxiliary task for QMIX to learn good representations. It has been demonstrated that our approach leads to a significant improvement in sample efficiency because each agent learns good representations and performs well, even when using the same amount of data within the same time step.
2. In contrast to previous studies, we perform experiments by reducing the time step by half for each scenario. Despite this constraint, the results show the superior performance of the proposed methodology compared to QMIX in 8 out of 11 scenarios from the StarCraft II micromanagement benchmark.

The remainder of this paper is organized as follows. Section 2 introduces related studies on MARL and sample-efficient RL. Section 3 provides the preliminary information necessary to understand this study. Section 4 presents a detailed explanation of the proposed method. Section 5 presents the results of the comparison and hyperparameter selection experiments for the 11 scenarios. Finally, Section 6 summarizes the conclusions of this study and suggests future research plans.

## Related works

### Multi-agent reinforcement learning

Inspired by the success of single-agent RL combined with deep learning in high-dimensional sensory inputs [30], many studies have been conducted to solve challenging cooperative tasks in multi-agent systems. The most naïve approach for a multi-agent system is independent Q-learning (IQL) [24], in which each agent learns individual action-value functions independently and does not rely on communication or coordination among agents during training. However, this decentralized approach has certain limitations, such as non-stationarity and spurious reward problems, because the behaviors of other agents influence the dynamics of the environment [25]. Since then, notable advances have been made in centralized approaches using joint learning algorithms to address these limitations. Value-decomposition network (VDN) [25] used a joint learning algorithm by decomposing the joint action-value function into the sum of individual agent action-value functions, which solves the spurious reward problem in perfectly independent learners. However, VDN ignores additional state information because it estimates a joint action-value function conditionally only on local observations per agent. To address these limitations, counterfactual multi-agent (COMA) [26] used a centralized critic to estimate a joint action-value function considering other agents’ observations and additional joint state information, whereas individual agents act in a decentralized manner conditioned on local observations.

Multi-agent deep deterministic policy gradient (MADDPG) [27] aligns with COMA in terms of a centralized critic paradigm, considering a continuous action space in competitive environments and building a centralized critic per agent. However, the aforementioned method assumes a linear relationship between agent actions. In complex multi-agent environments, joint action values are required to account for the nonlinear relationships between agent actions. QMIX addressed this limitation using a mixing network to design a joint action-value function with a complex nonlinear combination of observations per agent and additional state information. Moreover, QMIX is a highly sample-efficient off-policy algorithm that outperformed previous MARL methods in various scenarios of the StarCraft multi-agent challenge (SMAC) [29]. Therefore, in this study, we propose an approach to improve sample efficiency by adopting QMIX as the MARL method and integrating an auxiliary task.

### Sample-efficient reinforcement learning

Recently, methods have been proposed to improve the sample efficiency and representation capability of DRL in single-agent environments by adopting self-supervised representation learning. Contrastive unsupervised representations for reinforcement learning (CURL) [15] introduced a simple framework that combines contrastive learning and single-agent RL. CURL used soft actor-critic (SAC) [31] and Rainbow [32] for DRL and adopted momentum contrast (MoCo) [33] as an auxiliary task. Although CURL has been successful in high-dimensional image-based domains, it does not consider contextual properties, such as correlations among consecutive frames [16]. To address this limitation, masked contrastive learning for RL (M-CURL) [16] used a transformer [34] to utilize the temporal context in consecutive frames. DRL combined with contrastive learning improved sampling efficiency; however, it is computationally expensive because of the large amount of memory required to store many negative samples. Several methods have been developed to overcome this limitation and improve the sample efficiency without using negative samples. Self-predictive representation (SPR) [18] proposed a representation learning scheme for DRL that exploits a multistep forward dynamics model and a self-predictive objective. Mask-based latent reconstruction (MLR) [23] predicted the complete state representations from consecutive frames using spatiotemporal cube masking. Both methods [18, 23] showed that it was possible to train a highly expressive encoder without using large negative samples.

The above methods have been proposed to improve sample efficiency using an auxiliary task in single-agent problems. However, there has bee n no significant progress in research on methods to improve sample efficiency using an auxiliary task in multi-agent problems. Our method is somewhat similar to MLR but with a few differences. Our method is based on a multi-agent setting, whereas MLR is based on a single-agent setting. Furthermore, unlike MLR, which applies masking to the state in a fully observable Markov decision process (MDP), we apply random feature masking to the observation considering a partially observable MDP (POMDP). Therefore, in this study, we propose a method that integrates QMIX with a masked reconstruction task to improve sample efficiency.

## Preliminaries

### Dencentralized partially observable Markov decision process

MARL learns the joint policy *π* of agents through cooperative multi-agent sequential decision-making. MARL consists of a decentralized partially observable Markov decision process (Dec-POMDP) [35] as a tuple *T* = (*S*, *U*, *P*, *R*, *O*, *Z*, *n*, *γ*). S is the set of overall states of the environment, and *A* ≡ {1, …, *n*} represents the set of actions of each agent at each time step. Each agent selects an action from the action set to form a joint action *U* ≡ *U*^*n*^ set. *P* : *S* × *U* × *S* → [0, 1] denotes the transition probability distribution. All agents use the same reward function R:S×U→R, and *γ* represents the discount factor. We consider the partially observable scenario where each agent can obtain individual observations *Z* from observation *O* : *S* × *A* → *Z*. Each agent has an action-observation transition *τ*^*a*^ ∈ *T* ≡ (*Z* × *U*)* according to the policy *π*^*a*^(*u*^*a*^|*τ*^*a*^) : *T* × *U* → [0, 1]. The joint policy *π* has a joint action-value function $Q^{\pi }\left(s_{t},u_{t}\right)=E_{s_{t+1:\infty },u_{t+1:\infty }}[R_{t}|s_{t},\;u_{t}]$ and $R_{t}=\sum_{i=0}^{\infty }\gamma^{i}r_{t+i}$ is defined as the discounted return. The goal of MARL is to determine the joint policy *π* that maximizes the expectation of the defined discounted return *R*_*t*_ in a Dec-POMDP environment.

### Deep Q-learning

Deep Q network (DQN) [30] is a method for solving discrete action benchmarks that approximate the Q-function by combining off-policy Q-learning and neural networks, and it can be applied in multi-agent settings. The neural network *Q*_*θ*_, parameterized by *θ*, is defined by the action-value function $Q_{\theta }^{\pi }\left(s,\;u\right)=E_{s,u,s^{\prime }∼D}[R(s,u)+\gamma E_{u^{\prime }∼\pi }[Q_{\theta }^{\pi }(s^{\prime },u^{\prime })]]$. The policy *π* is learned by minimizing the following loss between the value predicted by *Q*_*θ*_ and the target value estimated by network $Q_{\theta^{-}}$ at the previous time step:

$$\begin{array}{c} L_{\theta }=E_{(s,u,s^{\prime })∼D}[{\left(Q_{\theta }(s,\;u)-(R(s,\;u)+\gamma \;\underset{u^{\prime }}{\text{max}}\;Q_{\theta^{-}}(s^{\prime },\;u^{\prime })\right)}^{2}], \end{array}$$ (1)

where *D* is the replay memory that stores tuple (*s*, *u*, *r*, *s*′). *θ*^−^ is copied periodically from *θ* of network *Q*_*θ*_ and is a fixed parameter of the target network when *Q*_*θ*_ updates several iterations.

### Bootstrap your own latent

BYOL is one of the self-supervised learning methods for learning useful representations from unlabeled data [28]. BYOL trains a neural network to predict a one-view representation of an unlabeled data sample from another representation of the same sample. The training process for BYOL includes both online and target networks. At each training iteration, only the online network is learned by minimizing the following loss $L_{\theta ,\xi }^{BYOL}$, which is a weighted sum of $L_{\theta ,\xi }$ and ${\tilde{L}}_{\theta ,\xi }$:

$$\begin{array}{c} L_{\theta ,\xi }≜{\left\|\bar{q_{\theta }}\left(z_{\theta }\right)-{\bar{z}}_{\xi }^{\prime }\right\|}_{2}^{2}=2-2\cdot \frac{\left\langle q_{\theta }\left(z_{\theta }\right),z_{\xi }^{\prime }\right\rangle }{{\left\|q_{\theta }\left(z_{\theta }\right)\right\|}_{2}\cdot {\left\|z_{\xi }^{\prime }\right\|}_{2}}\cdot , \end{array}$$ (2)

$$\begin{array}{c} L_{\theta ,\xi }^{BYOL}=L_{\theta ,\xi }+{\tilde{L}}_{\theta ,\xi }, \end{array}$$ (3)

where $z_{\xi }^{\prime }$ is the target projection from a target network and *q*_*θ*_(*z*_*θ*_) is the prediction from an online network. The target network’s parameters *ξ* are updated periodically during the training process by moving average of the online network’s parameters *θ*. The target network is updated using the following equation:

$$\begin{array}{c} \theta \leftarrow optimizer(\theta ,\nabla_{\theta }L_{\theta ,\xi }^{BYOL},\eta ), \end{array}$$ (4)

$$\begin{array}{c} \xi \leftarrow \tau \xi +(1-\tau )\theta , \end{array}$$ (5)

where *η* is a learning rate, set within the range of 0 to 1.

## Proposed method

In this Section, we describe the proposed method, M-QMIX, that combines QMIX with a masked reconstruction task. Compared to QMIX, the proposed method enables a more meaningful extraction of representations, allowing each agent to explore the optimal policy and improve sample efficiency.

QMIX [11] is a powerful MARL method that uses a joint action-value function to capture the interdependencies between agents, resulting in better performance than other techniques. The individual agent action-value functions are combined into a joint action-value function through a mixing network trained with value decomposition to maintain the optimal policy. The mixing function ensures that the joint action-value function satisfies a monotonicity constraint that guarantees the optimality of the learned policy and preserves the optimal policy at the weights of the mixing network. QMIX becomes an adaptable approach for various MARL tasks using a centralized action-value function that satisfies the monotonicity constraint.

**Fig 1:**
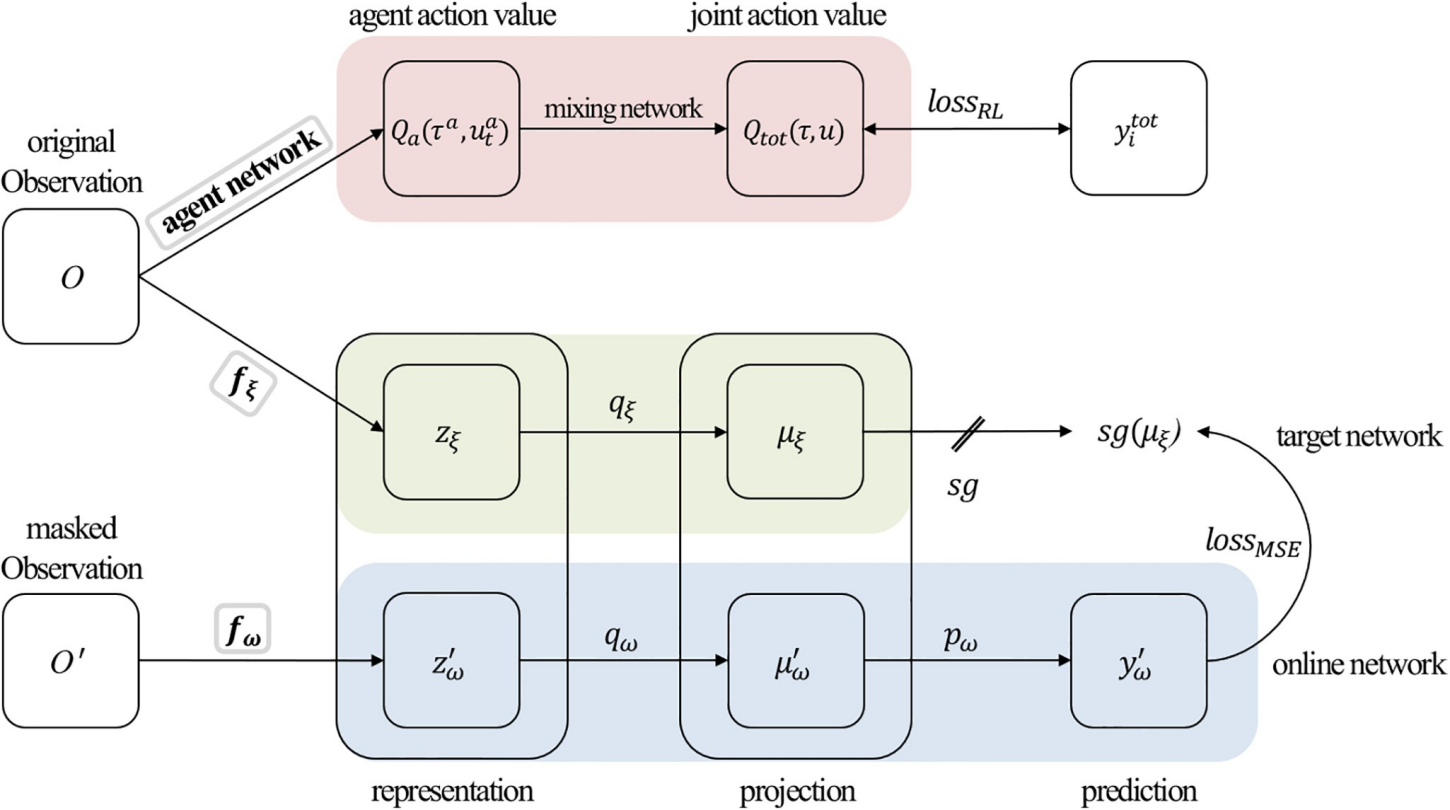
Overall architecture of the proposed method, which combines QMIX with a masked reconstruction task. Masked reconstruction task consists of a target and online network. The gray boxes represent the three recurrent networks.

Inspired by a solution to the limitations of sample inefficiency by combining self-supervised learning with single-agent RL for learning state representations [23], we used a masked reconstruction task in QMIX as an auxiliary task. Fig 1 shows the overall architecture of M-QMIX. The proposed method uses three networks: agent, online, and target. The encoders of the three networks have a structure consisting of two feedforward layers and one recurrent layer composed of a GRU cell [36]. The entire network is trained in an end-to-end manner. Next, we introduce masking and illustrate the training processes for these three networks.

### Masking samples

We randomly sample *T* observations from the replay buffer. Sampled observations are denoted as *O* = (*o*_1_, *o*_2_, *o*_3_, *o*_4_, ⋯, *o*_*T*_). To perform the masked reconstruction task, we define *M* = (*M*_1_, *M*_2_, *M*_3_, *M*_4_, *M*_5_, ⋯, *M*_*T*_), using masking ratio *r*_*m*_ ∈ [0, 1], which is a hyperparameter. If *r*_*m*_ = 0.2, 20 percent of the values in the *M*_*i*_ matrix are set to the zero vector, and the remaining 80 percent are set to one vector. The modified observation values are denoted as $o_{t}^{\prime }$. $O^{\prime }=\left(o_{1}^{\prime },o_{2}^{\prime },o_{3}^{\prime },o_{4}^{\prime },\cdot \cdot \cdot ,o_{T}^{\prime }\right)$ denotes the refined *O*, where $o_{t}^{\prime }=o_{t}⊙M_{t\;}\forall t\in \{1,2,\ldots ,T\}$.

### Agent network

**Fig 2:**
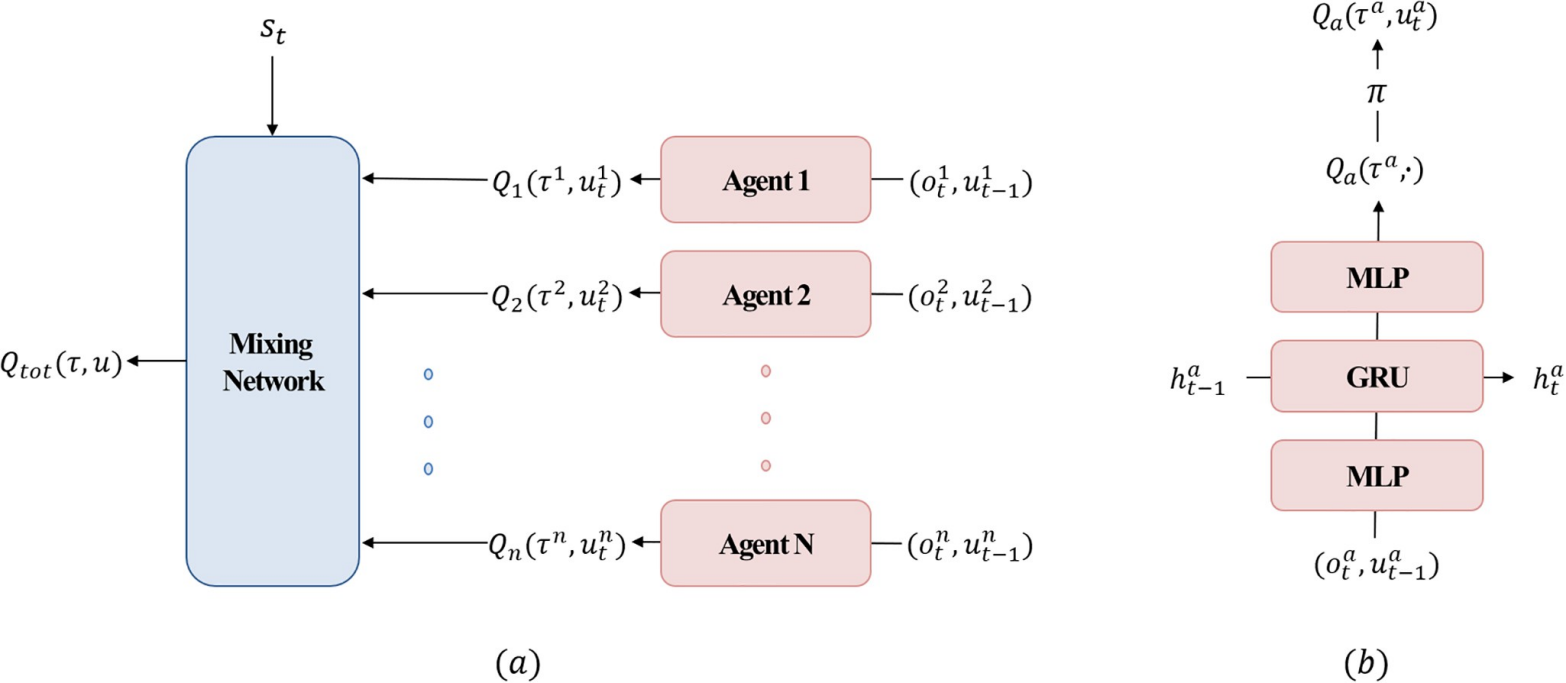
(a) Overall framework of QMIX. The output values obtained from each agent network are monotonically mixed to generate a joint action value function. (b) Agent network architecture. The network takes the current observation and the last action of an individual agent as inputs and outputs the corresponding Q-value for each agent.

The agent network serves as the primary component for extracting the Q-values of individual agents. Fig 2 shows the overall architecture of QMIX. The learning process comprises two stages. The first stage involves using the existing agent network, which is implemented as a deep recurrent Q-network (DRQN) [37], for each agent *a*. Specifically, the agent network takes the current observation $o_{t}^{a}$ and the last action $u_{t-1}^{a}$ as inputs for the agent and generates a Q-value $Q_{a}\left(\tau^{a},u_{t}^{a}\right)$ for each agent. This process is illustrated in Fig 2b. In the second stage, the Q-values for each agent and additional state information *s*_*t*_ are monotonically mixed as inputs to the mixing network and generate a joint action value function *Q*_*tot*_(*τ*, *u*). This process is illustrated in Fig 2a. The following loss is calculated using the produced *Q*_*tot*_(*τ*, *u*):

$$\begin{array}{c} L_{RL}\left(\theta \right)=\sum_{i=1}^{b}\left[{\left(y_{i}^{tot}-Q_{tot}\left(\tau ,u,s;\theta \right)\right)}^{2}\right], \end{array}$$ (6)

where *b* represents the batch size of the transitions sampled from the replay buffer. The target values are calculated using the following equation:

$$\begin{array}{c} y_{i}^{tot}=r+\gamma {max}_{u^{\prime }}Q_{tot}\left(\tau^{\prime },u^{\prime },s^{\prime };\theta^{\prime }\right), \end{array}$$ (7)

where *θ*′ denotes the parameters of the target network used in the DRQN.

### Online and target network

Online and target networks, which are identical to the asymmetric architecture of BYOL [28], are specifically designed for a masked reconstruction task and exhibit a notable distinction in their input data. The online network uses masked data based on *r*_*m*_. However, the target network uses the original data to obtain the ground truth. The online network consists of three stages, an encoder *f*_*ω*_, a projector *q*_*ω*_, and a predictor *p*_*ω*_. The target network uses the same network structure as the online network but with different parameters *ξ*. To update the target network parameters *ξ* using a momentum-based moving average of the online network parameters *ω*, we use the following equation:

$$\begin{array}{c} \xi =m\xi +(1-m)\omega , \end{array}$$ (8)

where *m* ∈ [0, 1] denotes the momentum value which determines the contribution of the online network parameters to this update.

Upon receiving a transition set that has been sampled up to the batch size from the replay buffer, a masked version of the original data, denoted by *O*′, is created in accordance with the masking ratio *r*_*m*_. The online network then uses the masked data as input to produce a representation feature $z_{\omega }^{\prime }≜f_{\omega }\left(o_{t}^{\prime }\right)$, which is subsequently projected onto $\mu_{\omega }^{\prime }≜q_{\omega }\left(z_{\omega }^{\prime }\right)$. Concurrently, the target network outputs a corresponding representation feature $z_{\xi }\;≜f_{\xi }\left(o_{t}\right)$ and projection $\mu_{\xi }≜q_{\xi }\left(z_{\xi }\right)$. After the execution of these steps, the online network generates a predicted value $y_{\omega }^{\prime }≜p_{\omega }\left(\mu_{\omega }^{\prime }\right)$. The target projection *μ*_*ξ*_, which is derived from the target network served as the correct answer. To calculate the mean squared error (MSE) loss, we normalize $y_{\omega }^{\prime }$ and *μ*_*ξ*_ to ${\bar{y}}_{\omega }^{\prime }≜y_{\omega }^{\prime }/{\left\|y_{\omega }^{\prime }\right\|}_{2}$ and ${\bar{\mu }}_{\xi }≜\mu_{\xi }/{\left\|\mu_{\xi }\right\|}_{2}$ using *l*_2_ normalization. The MSE and total losses are calculated as follows:

$$\begin{array}{c} L_{MSE}={\left\|{\bar{y}}_{\omega }^{\prime }-{\bar{\mu }}_{\xi }\right\|}_{2}^{2}, \end{array}$$ (9)

$$\begin{array}{c} L_{total}=L_{RL}+L_{MSE}. \end{array}$$ (10)

The total loss is obtained by summing *L*_*RL*_ from QMIX and *L*_*MSE*_ from the masked reconstruction task, after which the parameters of the online and agent networks are updated to minimize the loss. Notably, M-QMIX is a study to integrate QMIX with a masked reconstruction task, facilitating the learning a more effective representation of the encoder in QMIX.

## Experiments

Real-time strategy (RTS) games have received considerable attention as challenging benchmarks for RL environments. In this study, we chose the StarCraft II micromanagement benchmark [29], a popular RTS game, as a testbed to evaluate the impact of incorporating a masked reconstruction task on the sample efficiency of QMIX. The StarCraft II micromanagement benchmark involves using small-scale combat situations within the StarCraft II game for reinforcement learning assessment. Agents engage in intricate micro-level tactics to command units effectively. This benchmark includes scenarios classified as super hard, hard, or easy, each evaluating agent strategies and collaborative capabilities according to the specific complexity [29]. For the main experiments, we selected a set of 11 scenarios from the StarCraft II micromanagement benchmark, comprising three super hard, three hard, and five easy scenarios. Furthermore, we conducted experiments involving a half reduction in the time steps in each of the pre-existing scenarios, except for super hard scenarios. In these super hard scenarios, we increased the time steps by an additional 50K because of the task’s extreme difficulty. However, even with 2.5M time steps, it still doesn’t represent a substantial number in the StarCraft II micromanagement benchmark. The purpose of these modifications was to empirically validate the improved sample efficiency of the proposed method compared to QMIX. Our results underscore the efficacy of our approach in optimizing resource utilization and enhancing training efficiency by successfully achieving more desirable outcomes with reduced training duration. We conducted a comparative analysis with QMIX, using the average score obtained from eight random seeds as the basis for comparison, to evaluate the further improved sample efficiency of M-QMIX.

We selected one super hard, one hard, and one easy scenario for the hyperparameter selection experiments, which showed a clear performance difference between QMIX and M-QMIX in the main experiment. We used the average values obtained from three random seeds for comparative analysis to validate the effect of the momentum value used for the momentum update and masking ratio on the masked reconstruction task. Specifically, the timesteps used for each scenario remained consistent with those of the main experiment, ensuring a fair and consistent basis for comparison throughout the evaluation process.

### Main results

In all the scenarios, we hypothesized that the agent’s acquisition of a meaningful representation derived from the data elicited through its interactions with the environment would facilitate the exploration of optimal policies, even when confronted with limited data. We conducted an empirical investigation by using a masked reconstruction task as an auxiliary task to validate our hypotheses. Furthermore, given the constraints posed by limited data availability, we chose to use only half of the time steps used in previous studies [29].

**Fig 3:**
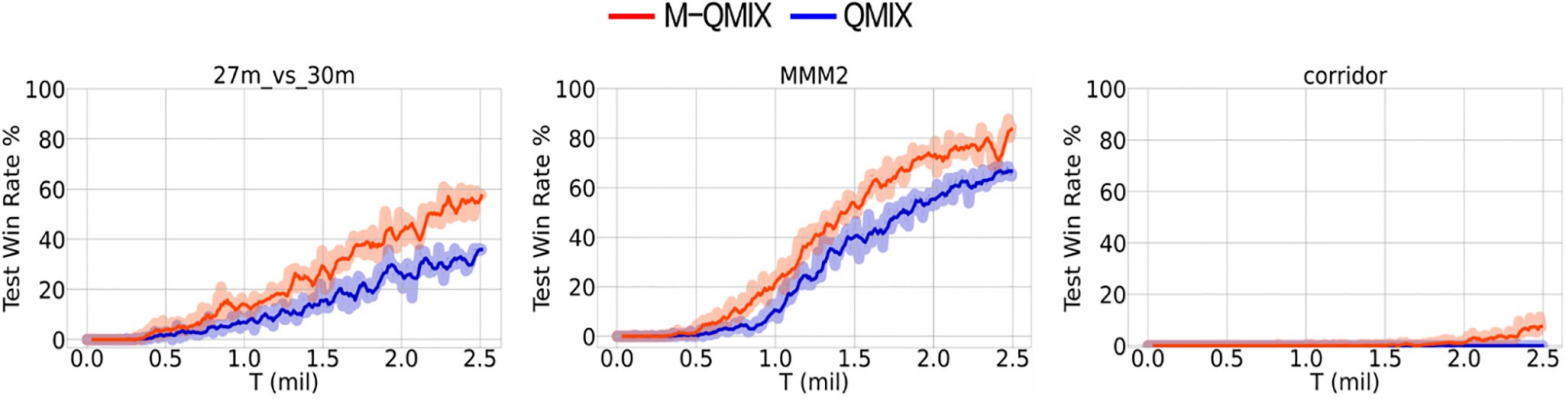
Comparison between M-QMIX and QMIX on all super hard maps.

The super hard scenario consists of three distinct scenarios: MMM2 with an identical number of enemy and allied units, 27m_vs_30m with an unidentical number of enemy and allied units, and a corridor with an unidentical number of enemy and allied units and complex terrain. Fig 3 illustrates the results of QMIX and M-QMIX for all the super hard scenarios. The benefits associated with the integration of the masked reconstruction task as an auxiliary task were demonstrated by a superior performance compared to QMIX on all super hard maps, requiring more exploration. In particular, despite the small number of allied units on the 27m_vs_30m map, it showed the largest performance margin between M-QMIX and QMIX. Furthermore, M-QMIX outperformed QMIX after 700K training steps on both the 27m_vs_30m map and the MMM2 map. On the corridor map, QMIX converges to a win rate of zero, whereas M-QMIX achieves a win rate close to ten percent.

**Fig 4:**
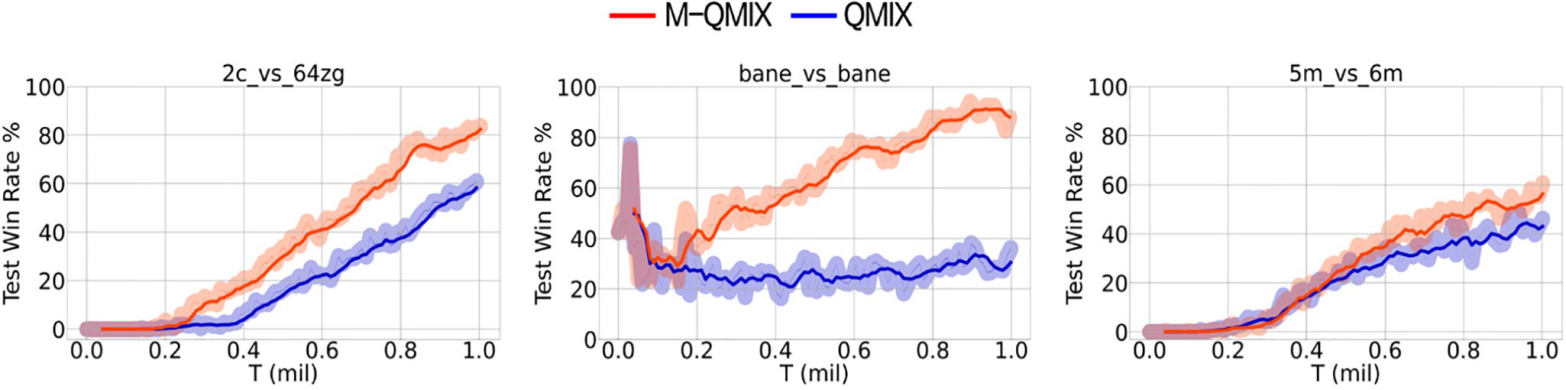
Comparison between M-QMIX and QMIX on all hard maps.

The hard scenario consists of three distinct scenarios: 2c_vs_64zg with a large unit gap, bane_vs_bane with identical numbers of enemy and allied units, and 5m_vs_6m with unidentical numbers of enemy and allied units. Fig 4 shows the results for all hard scenarios. For all hard maps, M-QMIX outperformed QMIX by a large margin. The largest performance margin between M-QMIX and QMIX was observed in the bane_vs_bane map, which achieved a significantly higher win rate within a relatively short training step. Moreover, despite both the 2c_vs_64zg map and the 5m_vs_6m map having fewer allied units, M-QMIX outperformed QMIX after 300K and 400K training steps, respectively.

**Fig 5:**
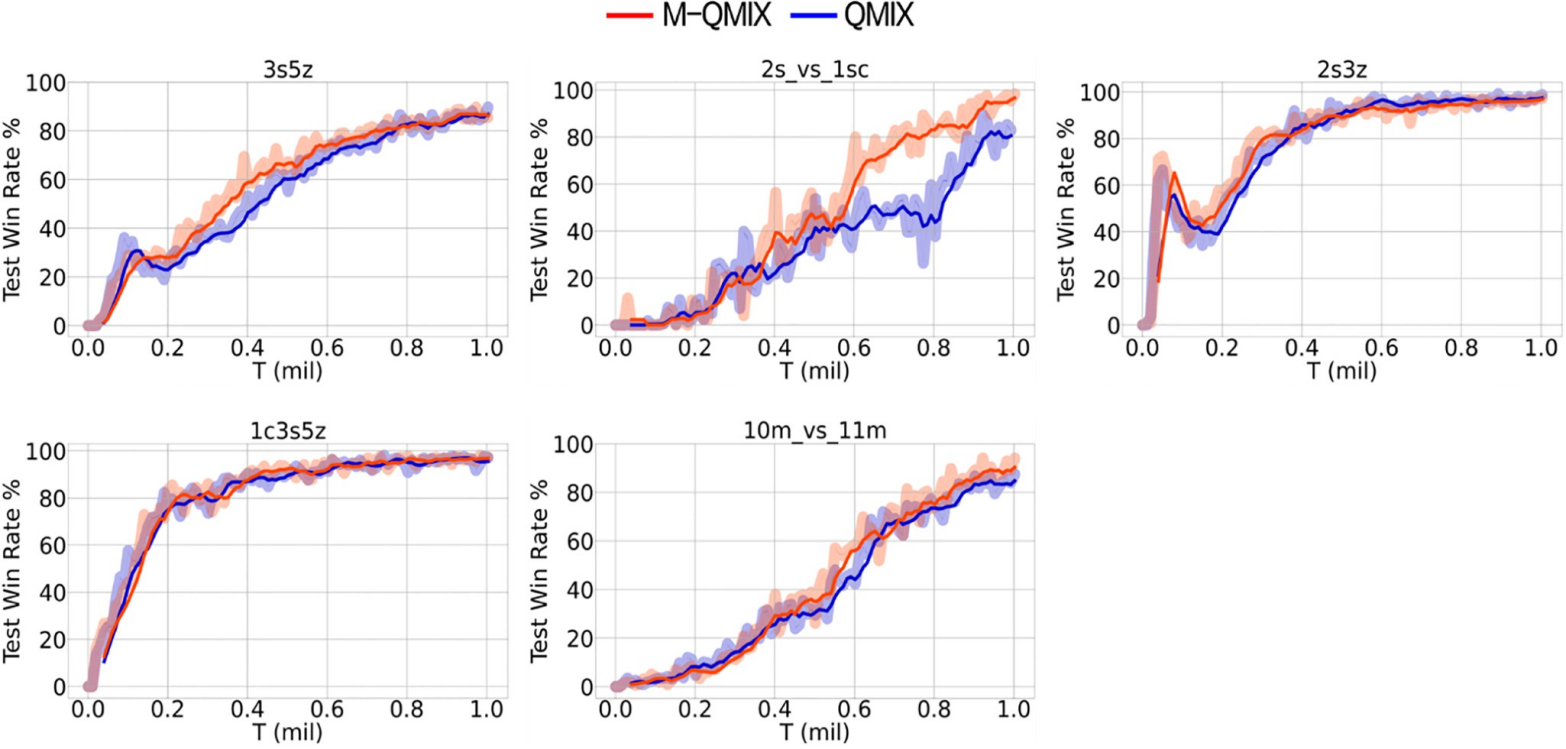
Comparison between M-QMIX and QMIX on all easy maps.

The easy scenario consists of five scenarios: 1c3s5z, 2s3z, and 3s5z with an identical number of enemies and allied groups, 2s_vs_1sc and 10m_vs_11m with an unidentical number of enemies and allied groups. Fig 5 shows the results for all easy scenarios. In the 2s_vs_1sc map, we can observe that M-QMIX outperformed QMIX by a large margin. Furthermore, on the 3s5z map, M-QMIX converges at a higher win rate faster than QMIX. For the remaining maps, M-QMIX outperformed QMIX by a small margin; We reasoned this was because QMIX could perform well on its own in easy scenarios.

### Hyperparameter selection

**Fig 6:**
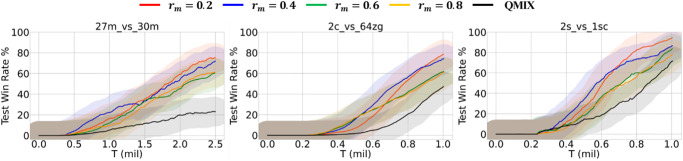
Performance of M-QMIX under different masking ratios.

As mentioned previously, for the two hyperparameter selection experiments, we selected three maps showing clear performance differences between QMIX and M-QMIX in the main experiment:27m_vs_30m (super hard map), 2c_vs_64zg (hard map), and 2s_vs_1sc (easy map). The masking ratio has a significant impact on the effectiveness of the masked reconstruction task. Therefore, we hypothesized that higher ratios would lead to inferior performance because of the excessive obscuring of information, making reconstruction challenging. We used a different percentage value *r*_*m*_ ∈ {0.2, 0.4, 0.6, 0.8} to mask the observation value. Fig 6 shows the results of M-QMIX for varying masking ratios. Regardless of the ratio, our method consistently outperformed QMIX, demonstrating the effectiveness of integrating the masked reconstruction task. We confirmed that setting *r*_*m*_ to 0.2 converged to the highest win rate in all scenarios, as hypothesized. However, increasing the value of *r*_*m*_ resulted in performance deterioration. This is because when a significant amount of information is obscured, it is difficult to restore it accurately, which adversely affects representation learning.

**Fig 7:**
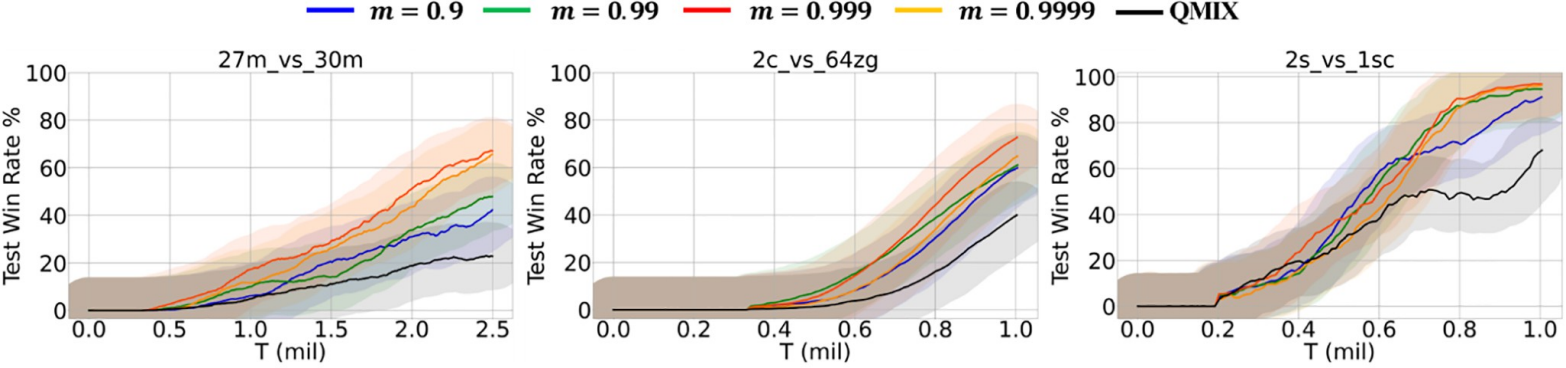
Performance of M-QMIX under different momentum values.

The momentum-based moving average used to update the target network can affect training stability depending on the chosen momentum value. Therefore, we hypothesized that using a higher momentum value would improve training stability and performance. Fig 7 shows the results of M-QMIX for various momentum values. We used a different value of momentum *m* ∈ {0.9, 0.99, 0.999, 0.9999}. Regardless of the assigned value, the proposed method consistently demonstrated superior performance compared to QMIX, underscoring the effectiveness of integrating the masked reconstruction task. As illustrated in Fig 7, when the momentum *m* is set to a small value, it results in greater learning instability and decreased performance. However, it was confirmed that increasing the value not only improves the performance because of the stabilization of training but also shows the highest performance when the momentum *m* is set to 0.999.

## Conclusion

The success of deep learning in single-agent systems has led to considerable research on solving challenging cooperative tasks in multi-agent systems. Furthermore, several studies have been proposed using representation learning as an auxiliary task to further improve the sample efficiency in single-agent systems. Extending the success of single-agent systems to multi-agent systems, we proposed M-QMIX, a study to further improve sample efficiency by using a masked reconstruction task as an auxiliary task for QMIX, a fundamental value-based methodology widely used in MARL methodologies. Our results in the StarCraft II micromanagement benchmark showed that the proposed M-QMIX not only outperformed QMIX over all super hard, hard, and easy scenarios but also demonstrated improved sample efficiency. Moreover, the main hyperparameter selection experiments of the proposed method not only yielded satisfactory metrics but also consistently outperformed QMIX, regardless of the selected hyperparameters.

In future studies, we aim to study new data augmentation methods for vector-shaped data, extending beyond the use of masking, to enhance the learning of meaningful representations. In the proposed methodology, masking was used to augment the vector-shaped data obtained from the StarCraft II micromanagement benchmark. However, recent research in the field of self-supervised learning has demonstrated improved performance by leveraging a diverse range of data augmentations. Given these findings, we anticipate that by developing suitable strategies for data augmentation, we can not only achieve effective representation learning by integrating multiple data augmentations but also improve the sample efficiency of our method. This will open up promising possibilities for advancing the state-of-the-art in the analysis and learning of vector-shaped data.
